# Acetic Acid Assisted Crystallization Strategy for High Efficiency and Long‐Term Stable Perovskite Solar Cell

**DOI:** 10.1002/advs.201903368

**Published:** 2020-01-23

**Authors:** Yong Li, Junwei Shi, Jianghui Zheng, Jueming Bing, Jianyu Yuan, Yongyoon Cho, Shi Tang, Meng Zhang, Yin Yao, Cho Fai Jonathan Lau, Da Seul Lee, Chwenhaw Liao, Martin A. Green, Shujuan Huang, Wanli Ma, Anita W. Y. Ho‐Baillie

**Affiliations:** ^1^ Australian Centre for Advanced Photovoltaics School of Photovoltaic and Renewable Energy Engineering University of New South Wales Sydney New South Wales 2052 Australia; ^2^ Jiangsu Key Laboratory for Carbon‐Based Functional Materials and Devices Institute of Functional Nano & Soft Materials (FUNSOM) Soochow University Suzhou 215123 China; ^3^ Electron Microscope Unit Mark Wainwright Analytical Centre The University of New South Wales Sydney New South Wales 2052 Australia; ^4^ School of Engineering Macquarie University Sydney New South Wales 2109 Australia; ^5^ John Hooke Chair of Nanoscience School of Physics Faculty of Science The University of Sydney Sydney NSW 2006 Australia

**Keywords:** crystal engineering, perovskites, photovoltaic, stability

## Abstract

Improving the quality of perovskite poly‐crystalline film is essential for the performance of associated solar cells approaching their theoretical limit efficiency. Pinholes, unwanted defects, and nonperovskite phase can be easily generated during film formation, hampering device performance and stability. Here, a simple method is introduced to prepare perovskite film with excellent optoelectronic property by using acetic acid (Ac) as an antisolvent to control perovskite crystallization. Results from a variety of characterizations suggest that the small amount of Ac not only reduces the perovskite film roughness and residual PbI_2_ but also generates a passivation effect from the electron‐rich carbonyl group (C=O) in Ac. The best devices produce a PCE of 22.0% for Cs_0.05_FA_0.80_MA_0.15_Pb(I_0.85_Br_0.15_)_3_ and 23.0% for Cs_0.05_FA_0.90_MA_0.05_Pb(I_0.95_Br_0.05_)_3_ on 0.159 cm^2^ with negligible hysteresis. This further improves device stability producing a cell that maintained 96% of its initial efficiency after 2400 h storage in ambient environment (with controlled relative humidity (RH) <30%) without any encapsulation.

## Graphical abstract

Acetic acid (Ac) is used as an antisolvent for preparing perovskite films with excellent optoelectronic properties. Ac is found to not only reduce perovskite film roughness and residual PbI_2_ but also generate a passivation effect from the electron‐rich carbonyl group. The best 0.159 cm^2^ devices produce efficiencies of 22.0% for Cs_0.05_FA_0.80_MA_0.15_Pb(I_0.85_Br_0.15_)_3_ and 23.0% for Cs_0.05_FA_0.90_MA_0.05_Pb(I_0.95_Br_0.05_)_3_.

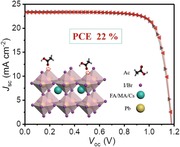

Organic–inorganic hybrid perovskite has become a rising star as a semiconductor material owing to its superb optoelectronic properties and its versatility in device applications. The high‐performance perovskite materials are not only applied to photovoltaics,^1^, ^2^, ^3^, ^4^, ^5^, ^6^, ^7^, ^8^ but also light‐emitting diodes (LEDs),^9^, ^10^, ^11^, ^12^, ^13^, ^14^ transistor,^15^, ^16^, ^17^ lasers,^18^, ^19^ and other optoelectronic applications.^20^, ^21^ To date, the power conversion efficiency (PCE) of the state‐of‐the‐art perovskite solar device has been certified to be 24.2%, higher than those of CdTe, CIGS, organic solar cells (OPV), dye‐sensitized solar cells (DSSCs), and quantum dot solar cells.^22^

It is believed that perovskite film quality is crucial to their optoelectronic properties. Many approaches have been employed to obtain a high‐quality perovskite microcrystalline film with fewer unwanted phases and defects. Different fabrication methods have been reported such as one‐step spin‐coating, two‐step sequential deposition and dual‐source vapor deposition, etc.^23^ The antisolvent method has been well proven for one‐step deposition and has become quite a universal method for obtaining a high‐quality film since it was first introduced in 2015.^24^ The antisolvent method facilitates rapid nucleation for subsequent grain growth producing continuous and homogeneous film after a short annealing. However, some unwanted phase or impurity residues can still remain on the surface generating charge recombination hindering the device performance and long‐term stability. Bromide and iodine vacancies have been reported to be the main defects in the perovskite thin film^25^, ^26^, ^27^ and excessive PbI_2_ at the surface or at the grain boundaries were also reported to generate undesirable hysteresis and instability.^28^, ^29^, ^30^ There remains a challenge to better control perovskite film crystallization for highly uniform grains without unwanted phase and defects. Using additives in the perovskite precursor is one of the effective means to reduce defects and to modify film morphology. For example, acids such as HCl or HI,^31^ 5‐ammoniumvaleric acid (5‐AVA)^32^ or organic material such as 1,8‐diiodooctane (DIO)^33^ and [6,6]‐Phenyl‐C_61_‐butyric acid methyl ester (PCBM)^34^ and even H_2_O^35^, ^36^ have been used and have been shown to modify the perovskite film enhancing device performance. The use of additive in antisolvent solution (normally chlorobenzene, CB) has been reported to be effective in modulating perovskite crystallization and in passivating the perovskite film. For example, Bi et al. introduced poly (methyl methacrylate) (PMMA) as an additive in the antisolvent solution which acts as a template for (FAI)_0.81_(PbI_2_)_0.85_(MAPbBr_3_)_0.15_ perovskite growth producing the best cell with a certified PCE of 21.02%.^37^ Fullerene and its derivative (α‐bis‐PCBM) were introduced by Zhang et al. which was reported to fill the vacancies and grain boundaries of perovskite film, as well as passivating Pb^2+^ antisite defects, enlarging the perovskite grain size and improving charge‐carrier separation and transport.^38^ The best (FAI)_0.81_(PbI_2_)_0.85_(MABr)_0.15_(PbBr_2_)_0.15_ perovskite device has an improved efficiency (20.8%) and stability (less than 10% efficiency drop after 44 d of storage in ambient air with 40% RH). Wang et al. added phenylalkylamine as the passivation molecules for FAPbI_3_ perovskite achieving a PCE of 19.2% with better stability (remain unchanged for > 2800 h of storage under air exposure with 50 ± 5 RH%).^39^ Yang et al. used HI as an additive in the antisolvent and a PCE of 19.9% was obtained on the best device with enhanced photoluminescence (PL) lifetime.^40^ Li et al. used N2200 and PFN polymers as additives in chlorobenzene modifying MAPbI_3_ film morphology and producing a surface passivation layer enhancing cell efficiency (18.7%) and longevity (less than 15% efficiency drop after 35 d of storage in an ambient environment with 30–40% relative humidity).^41^ Recently, the using of 2D materials such as phenethylammonium iodide (PEAI),^27^ *n*‐hexyl trimethyl ammonium bromide^42^ and *n*‐hexylammonium bromide^43^ for passivating the perovskite surface also achieved great improvement in device performance with the best device achieving certified efficiency over 23% and with enhanced stability.

Here, we introduce acetic acid (Ac) as the additive for the antisolvent (chlorobenzene) solution process for the fabrication of mixed halides triple‐cation Cs_0.05_FA_0.80_MA_0.15_Pb(I_0.85_Br_0.15_)_3_ perovskite. Our results show that the additive helps to modify perovskite film morphology producing ultra‐uniform surface. 2D Grazing‐Incidence Wide‐Angle X‐ray Scattering (GIWAX) results show that residual PbI_2_ on perovskite surface was greatly reduced as a result of using Ac in antisolvent. Furthermore, X‐ray photoelectron spectroscopy (XPS) characterization shows that film quality improves due to carboxyl (C=O) passivation effect. Collectively, the combined benefits of this method produces smoother perovskite film with fewer defects and fewer nonperovskite phase, enabling the corresponding best perovskite solar cell to achieve a PCE of 22.0% for Cs_0.05_FA_0.80_MA_0.15_Pb(I_0.85_Br_0.15_)_3_, which is the highest (to the best of our knowledge) reported efficiency for band gap ≈1.6 eV perovskite (which contains Br ≈0.15) and the highest for cells fabricated using antisolvent additive engineering as well.

For perovskite film fabrication, triple cation mixed halide perovskite precursor solution in *N*,*N*‐dimethylformamide (DMF)–dimethyl sulfoxide (DMSO) mixed solvent (see the Experimental Section for details of precursor preparation) was deposited by one‐step spin‐coating method. **Figure** **1**A shows the chemical structures of CB and Ac. Figure 1B illustrates the one‐step spin‐coating deposition for fabricating the perovskite film. For antisolvent engineering, different contents of Ac (0, 2, 5, 8, and 10 v%) in CB solutions (labelled as Ac 0, Ac 2, Ac 5, Ac 8, and Ac 10) were used and the mixture was dripped on the perovskite film surface during the spin coating of the perovskite precursor. The as‐deposited films were annealed to completely remove the solvents and to complete perovskite crystallization. Figure 1C shows the possible location of Ac within the perovskite crystal illustrating electron‐rich carboxyl (C=O) groups filling the halide vacancies of the undercoordinated Pb atoms.^24^, ^25^, ^27^

**Figure 1:**
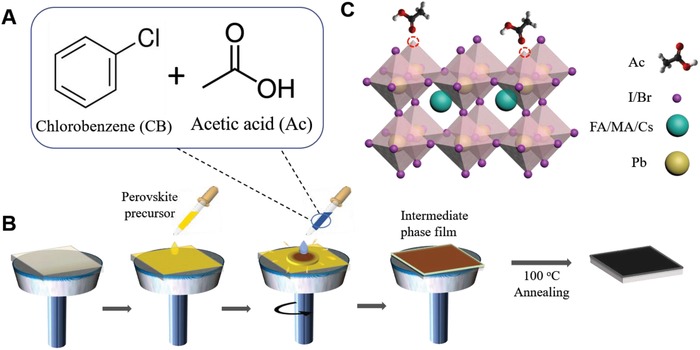
Acetic acid (Ac) assisted perovskite fabrication strategy. A) Chemical structures of chlorobenzene and Ac. B) Schematic illustration of the Ac assisted crystallization process. C) Passivation of dangling bonds by Ac in the perovskite crystalline film.

The left column of **Figure** **2** shows the top‐view scanning electron microscopy (SEM) images of the perovskite film fabricated by using a varied amount of Ac. Histograms inserted shows distribution of grain size. As Ac content increases, grain size distribution narrows, e.g., Ac 5 and Ac 8 treated films. Atomic force microscopy (AFM) was employed to examine the perovskite film topography, in 2D (results in middle column in Figure 2) and 3D (Figure S1, Supplementary information). Surface roughness RMS (root‐mean‐squared) values of each film are also inserted. Again, the surface becomes smoother with Ac and is most uniform at Ac 8. Smoother and more uniform perovskite film is beneficial for better charge transport resulting in higher photovoltaic performance. Similar findings were reported by Zhang et al.^44^ and Zhao et al.^45^ who found that the Ac in perovskite precursor improves CH_3_NH_3_PbI_3_ perovskite crystallization process enhancing the film uniformity. When the Ac content is low, Ac assists in triggering heterogeneous nucleation for the perovskite precursor film, improving the perovskite film quality. However, film morphology becomes worse for the Ac 10 film. This is attributed to the acidic effect of Ac (acid dissociation constant Ka = 1.76 × 10^−5^ at 25 °C).^46^ That is, when Ac is over a certain amount, the acid becomes too strong corroding perovskite crystal resulting in worse coverage, as shown in Figure S2 (Supporting Information). The films appear “wrinkled” with higher concentration of Ac (e.g., at Ac 8) as shown in Figure S3 (Supporting Information). These wrinkles are more prominent with increasing Ac possibly come from the released compressive strain inside the perovskite layer.^47^ Nevertheless, these wrinkles can be beneficial which have been shown to boost device performance for mixed halide perovskite solar cell.^48^, ^49^

**Figure 2:**
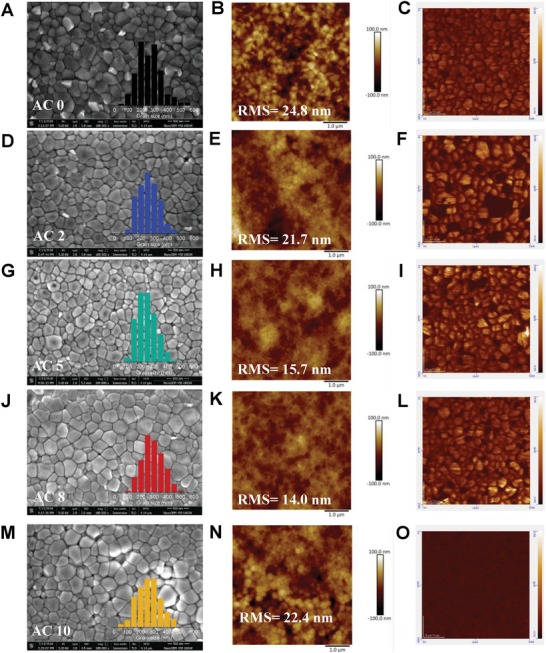
Cs_0.05_FA_0.80_MA_0.15_Pb(I_0.85_Br_0.15_)_3_ perovskite surface characterizations. Including scanning electron microscopy (SEM), atomic force microscopy (AFM), and conductive atomic force microscopy (c‐AFM) the perovskite films fabricated by Ac antisolvent treatment. A–C) without Ac, D–F) with Ac 2, G–I) with Ac 5, J–L) with Ac 8, and M–O) with Ac 10. Scale bars are 500 nm for SEM and 1.0 µm for AFM.

Conductive AFM (c‐AFM) was conducted to ascertain the conductivity of the perovskite film, with the results shown in Figure 2 (right column). The brighter color suggests more conductive grains, indicating the perovskite film treated by the Ac becomes more conductive benefitting carrier separation improving the photovoltaic performance.^50^ Kelvin probe force microscopy (KPFM) was used to characterize the spatial surface potentials of the perovskite films with and without Ac treatment (Figure S4, Supporting Information). Results show Ac treated film has more uniform distribution of surface potentials and represents the surface chemical and electrical characteristics are more homogeneous thus can be favorable for achieving better device performance and stability.^51^

Steady‐state PL and the absorbance of the perovskite films with different concentration of Ac treatment were measured. Results are shown in **Figure** **3**A, showing identical onset PL peaks (around 780 nm). This means Ac treatment and varying its concentration does not change significantly on the bandgap of our perovskite films, which is around 1.6 V, typical of film for this composition.^52^, ^53^ X‐ray diffraction (XRD) measurement was also carried out on FTO/*c*‐TiO_2_/*mp*‐TiO_2_/Cs_0.05_FA_0.80_MA_0.15_Pb(I_0.85_Br_0.15_)_3_ test structure. Results in Figure 3B show typical patterns of a black perovskite crystal with main diffraction peak at 14.1° for the (110) phase; peak at 12.7° for the (001) plane of hexagonal cubic PbI_2_. In the reference sample, Ac 0, there is a noticeable peak of PbI_2_ suggesting the presence of residual PbI_2_, while the PbI_2_ peak is suppressed with Ac treatment. The backscattered SEM (BS‐SEM) was also conducted on Ac 0 and Ac 8 samples which also show that residual PbI_2_ is greatly suppressed in Ac 8 film (Figure S5, Supporting Information). This can be explained by the two key chemical reactions as represented by Equations (1) and (2) for perovskite formation and PbI_2_ precipitation, respectively.^54^ As formamidinium iodide (FAI) is the major cation component in our mixed perovskite system, we only use the FAI in the equations for the purpose of illustration:

(1) $${\text{PbI}}_{2}+{\text{Ac}}^{-}+\;x\text{FAI}\to {\text{FAPbI}}_{3}+\text{FAAc}↑+\;\left(x-1\right)\text{FAI}↑$$

(2) $${\text{FAPbI}}_{3}\to \;\text{FAI}↑\;+\;{\text{PbI}}_{2}$$

**Figure 3:**
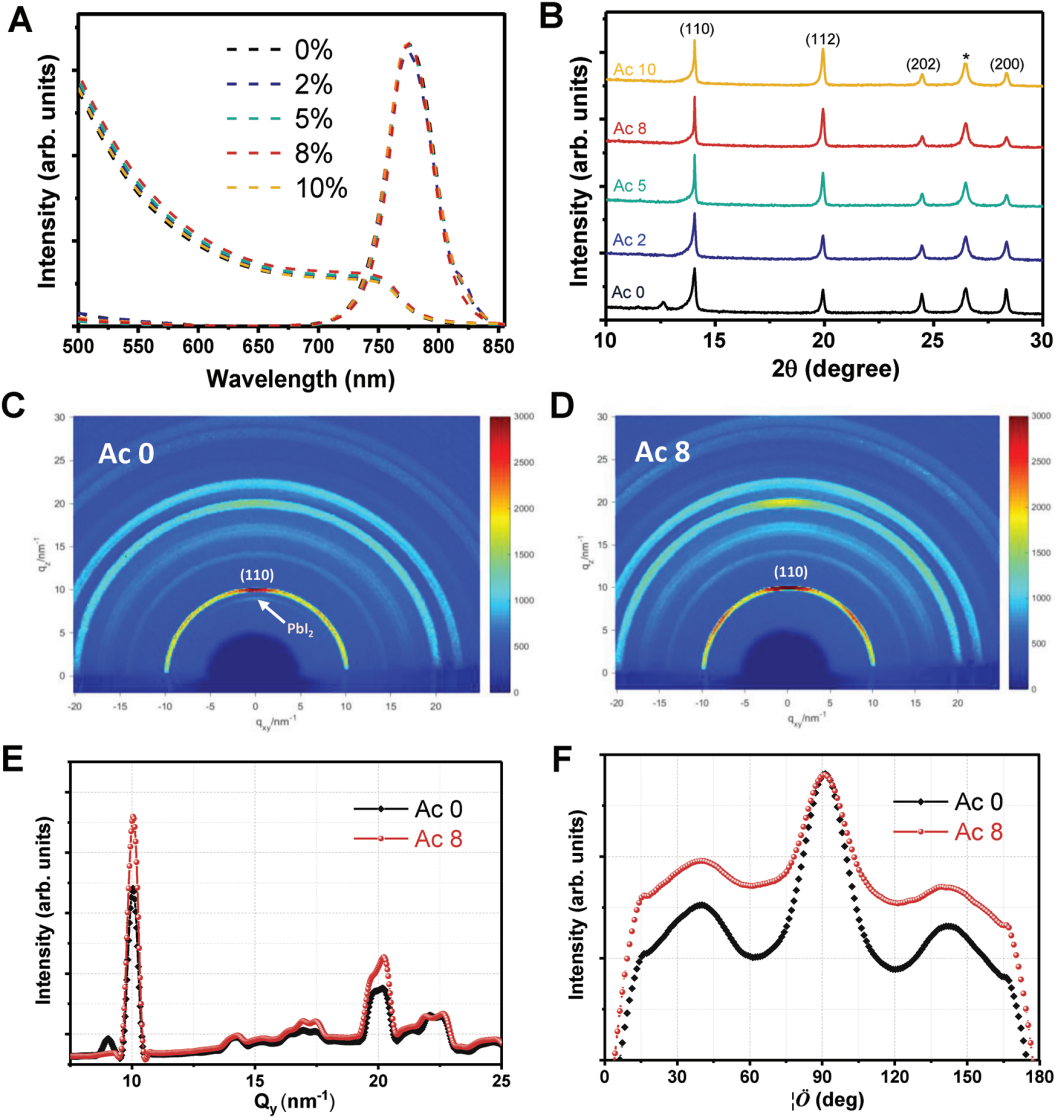
Cs_0.05_FA_0.80_MA_0.15_Pb(I_0.85_Br_0.15_)_3_ perovskite crystal characterizations. A) Absorbance and steady‐state PL of perovskite films. B) X‐ray Diffraction (XRD) patterns and C,D) 2D Grazing‐Incidence Wide‐Angle X‐ray Scattering (GIWAX) results for reference (Ac 0) and Ac treated (Ac 2 to Ac 8) perovskites. * represents FTO peak. E) Out‐of‐plane GIWAX diffraction line profile of Ac 0 and Ac 8 and F) radially integrated intensity plots along the *Q_y_* = 10 nm^−1^ ring, which represents the perovskite (110) plane.

As suggested by the thermal gravimetric analysis (TGA) from previous research,^44^ the generated Ac‐based salt in Equation (1) is thermal unstable and much more volatile than FAI. Therefore, the larger amount of residual FAI will hinder the second reaction Equation (2) resulting in less PbI_2_ being generated in Ac‐treated perovskite. Similar finding was reported by Zhang et al. who introduced Ac in the perovskite precursor and found that traces of PbI_2_ are greatly suppressed in the perovskite film because of the changes in growth kinetics.^44^

To further determine crystallinity and orientation of the perovskite film, GIWAX measurement was conducted on the Ac 0 and Ac 8 treated films. Results are shown in Figure 3C,D, respectively. Again, the PbI_2_ peak is suppressed in the AC 8 treated film (cf. Figure 3C,D). There is a stronger preference for the (110) plane in the Ac 8 film compared to the Ac 0 film. This is evident in the out‐of‐the‐plane line profile of the Ac 8 film shown in Figure 3E which has a stronger peak at *q* = 10 nm^−1^, corresponding to the (110) perovskite peak as *q* = 4πsin (θ)/λ. Figure 3F shows the azimuthally integrated scattering intensity of GIWAX pattern along the ring for *q* = 10 nm^−1^. Interestingly, Ac 8 film has stronger peaks for the two preferred orientations at the azimuth angles of around 40° and 142°. These findings show that Ac 8 film has better crystallinity with stronger preferred orientations than reference film without any antisolvent additive treatment.

In order to further explore the role of Ac in the perovskite film chemically, XPS was conducted on the Ac treated films. XPS spectra of the films in Figure S6 (Supplementary information) are identical suggesting no change in perovskite composition after Ac treatment. Elemental scans for Pb, I, C, and O were carried out under high resolution. Results are shown in **Figure** **4**. The characteristic Pb 4f_7/2_ and Pb 4f_5/2_ peaks at 138.7 and 142.8 eV respectively were blue shifted with increasing Ac by as much as 0.3 eV for Ac 8 film (Figure 4A). However, the positions of the peaks of Ac 10 film reverted, similar to those of the control (Ac 0) film. Regarding XPS for C 1s (Figure 4B), while the peaks of C—C remain at 284.8 eV for all the films, the C=O peaks were blue shifted with increasing Ac by as much as 0.4 eV for Ac 8 film. Meanwhile, O 1s peaks of Ac films were red shifted with increasing intensity with Ac treatment (Figure S6, Supporting Information). The Pb 4f peak shift, the C=O peak shift and the O 1s peak shift indicate that there is chemical interaction between the carboxyl (C=O) group in Ac and the Pb in the perovskite. To further show the interaction between Ac and PbI_2_, Fourier‐transform infrared spectroscopy (FTIR) was conducted on Ac solution and Ac mixed with PbI_2_ in DMSO and DMSO for reference. Results are shown in Figure 4C. The introduction of PbI_2_ into Ac causes a shift of the C=O stretching vibration bond from 1730 to 1712 cm^−1^. These results aid the explanation of the C=O and O 1s peaks shift in the XPS results. It has been previously reported that the carboxyl group (C=O) is a Lewis base site that can donate electron pair from C=O double bond to the under‐coordinated lead atoms in the perovskite,^37^, ^55^ thereby providing a passivating effect to the perovskite film.

**Figure 4:**
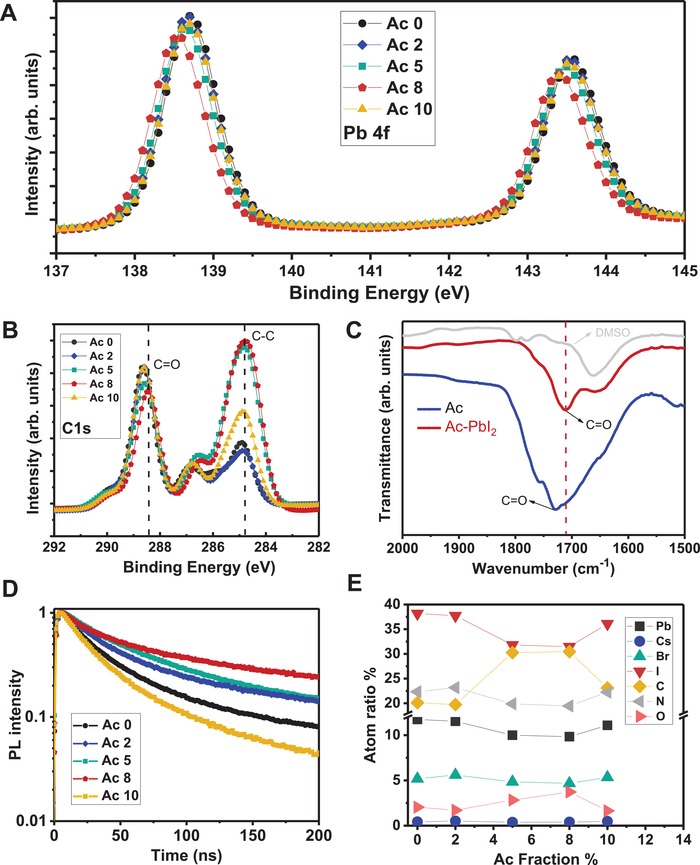
Understanding the Ac effect on perovskite. X‐ray photoelectron spectroscopy (XPS) result of A) Pb 4f and B) C1s with different Ac treatments. C) FTIR spectra of pristine Ac and of the Ac‐PbI_2_ adduct, prepared by mixing Ac with PbI_2_ in DMSO. Note FTIR spectra of DMSO (gray line) is also shown as background. D) The PL lifetime of perovskite film with different Ac treatment. E) Atoms ratio changes in perovskite films treated by different Ac concentrations.

To investigate the carrier dynamics of Ac treated perovskite film, we conducted time‐resolved photoluminescence (TRPL) decay measurements on Cs_0.05_FA_0.80_MA_0.15_Pb(I_0.85_Br_0.15_)_3_/glass test structure. Results are shown in Figure 4D and the effective lifetimes calculated are summarized in Table S1 (Supporting Information). It is suggested that effective lifetimes of the perovskite films increase with Ac until Ac 8. This trend is in accordance with the trend observed from chemical characterizations with the best passivation at Ac 8. However, again, as Ac concentration exceeds a certain amount (e.g., for Ac 10), the passivation is less effective as the additive becomes more acidic deteriorating the perovskite film resulting in poor morphology. Figure 4E shows the atomic ratios (%) (determined from XPS) of the elements present in the Cs_0.05_FA_0.80_MA_0.15_Pb(I_0.85_Br_0.15_)_3_ perovskite films processed with different concentrations of Ac in the antisolvent. The I:Pb ratios for the films are also calculated and summarized in Table S2 (Supporting Information). There is a noticeable reduction in the proportion of Pb and I in the film when Ac concentration is around Ac 5–8 v%. These results in line with the evident reduction of PbI_2_ in the Ac treated films as observed under XRD, GIWAX, and BS‐SEM characterizations.

To verify the benefits of Ac additive antisolvent treatment, we fabricated solar cells with the structure of FTO/compact TiO_2_ (*c*‐TiO_2_)/mesoporous TiO_2_ (*mp*‐TiO_2_)/Cs_0.05_FA_0.80_MA_0.15_Pb(I_0.85_Br_0.15_)_3_/Spiro‐OMeTAD/Au (illustrated in **Figure** **5**A and cross‐sectional SEM shown in Figure 5B).

**Figure 5:**
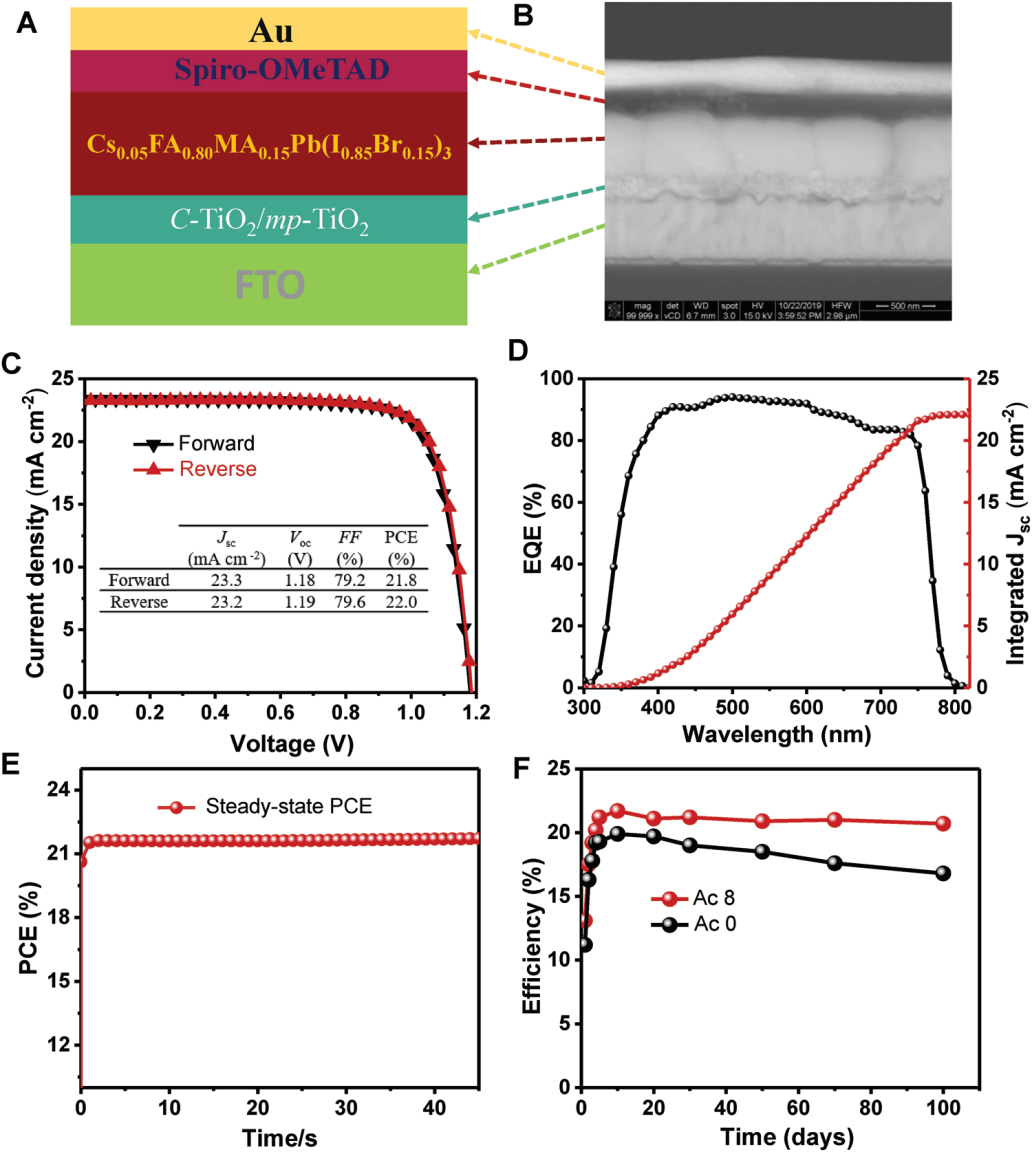
Fabricated Cs_0.05_FA_0.80_MA_0.15_Pb(I_0.85_Br_0.15_)_3_ perovskite device. A) Illustrated structure, B) cross‐section under backscattered SEM, C) forward and reverse scanned current density–voltage (*J–V)* curves of the best device measured under AM 1.5G solar irradiation at 100 mW cm^−2^, D) the corresponding external quantum efficiency (EQE) spectra. E) The corresponding steady‐state PCE under continuous illumination. F) Efficiency of an Ac 0 reference device and an Ac 8 device after 100 d (2400 h) of storage in an ambient atmosphere with RH <30%.

Performance of the typical Ac additive antisolvent treated Cs_0.05_FA_0.80_MA_0.15_Pb(I_0.85_Br_0.15_)_3_ perovskite solar cells are shown in Figure S7 (Supporting Information) and listed in Table S3 (Supporting Information). Distribution of cell parameters for each group of cells (20 in each group) with different levels of Ac treatment is shown in Figure S8 (Supporting Information). Results show that device performance increases with Ac concentration up until Ac = 8 v%. The best cell was fabricated with Ac 8 treatment and produced a steady‐state PCE of 21.7% at 0.99 V (Figure 5E) or a PCE of 22.0% under reverse scan (Figure 5C and table in the inset), a *J*_sc_ of 23.2 mA cm^−2^, a *V*_oc_ of 1.19 V, and an FF of 79.6% with negligible hysteresis. The corresponding external quantum efficiency (EQE) spectra of the best Cs_0.05_FA_0.80_MA_0.15_Pb(I_0.85_Br_0.15_)_3_ device was also measured and shown in Figure 5E and the integrated current density from the perovskite device is 22.1 mA cm^−2^ which agree with the *J*_sc_ values from the champion efficiency. The reference Ac 0 cell on the other hand only delivered an efficiency of 19.1% with a *J*_sc_ of 22.9 mA cm^−2^, a *V*_oc_ of 1.11 V, and an FF of 75.4%. The enhanced *J*_sc_ and FF are likely due to the better film morphology (Figure 2) and crystallinity (Figure 3) while enhanced *V*_oc_ is mainly due to Ac passivation effect consistent with the trend observed in TRPL result.

With regard to perovskite film stability, the Figure 5F and Figure S10 (Supporting Information) shows Ac 8 complete solar device is much more stable than Ac 0 device maintaining its initial PCE after 100 d (2400 h) of storage in ambient environment (with controlled relative RH of <30%) without any encapsulation (Figure S10, Supporting Information). Similarly, as‐deposited perovskite films with different levels of Ac treatment (**Figure** **6**A,B) are stored in ambient atmosphere at room temperature and RH of 50–70% with indoor light for over two months (temperature and RH are recorded and shown in Figure 6C). It is evident that Ac 8 perovskite is more stable than other films (darkest color after 70 d exposure). This is likely to be due to less unwanted phases and lower defects density. To compare the thermal stability of perovskite devices with and without Ac treatment under ambient, thermal stress test was conducted at 60 °C and RH of 40–60% (Figure 6E) for over 200 h. Results of the Ac0 and Ac8 devices show that Ac treated device has better thermal stability.

**Figure 6:**
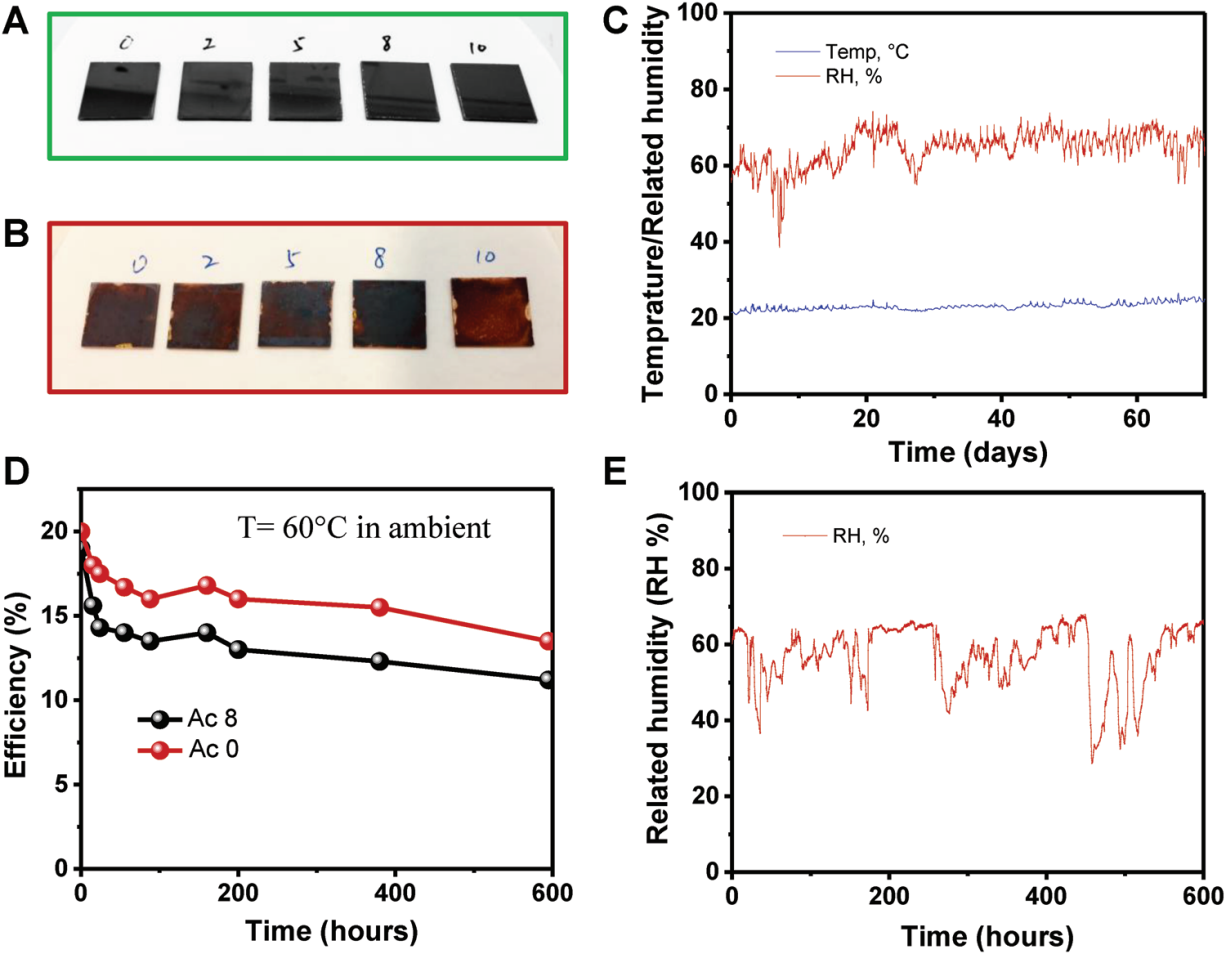
Stability test for perovskite film and device without encapsulation. Perovskite film with different levels of Ac treatments A) before and B) after 70 d in ambient, with C) temperature and related humidity recorded for the film test. D) Thermal stability test of an Ac 0 reference device and an Ac 8 solar cell for over 200 h with E) RH recorded for the thermal stress test.

In order to confirm the universality of Ac assisted crystallization for different bandgap perovskite system, we further fabricated lower bandgap (≈1.5 eV) Cs_0.05_FA_0.90_MA_0.05_Pb(I_0.95_Br_0.05_)_3_ perovskite solar cell. As shown in Figure S11 (Supporting Information), very high efficiency of 23.0% was achieved with a *V*_oc_ of 1.15 V, *J*_sc_ of 24.31 mA cm^−2^, and an extremely high FF of 82.3%, this is one of the highest reported efficiencies so far.

In summary, we introduced a unique and simple method to fabricate high‐quality perovskite film by using mixed Ac and chlorobenzene as the antisolvent in one‐step perovskite deposition. Our result shows that 8% of Ac can improve the perovskite film properties, including morphology and reduced residual PbI_2_ as well as reduction of defects provided by chemical passivation from the carboxyl (C=O) group in the Ac. PCE of 22.0% was achieved for the champion Cs_0.05_FA_0.80_MA_0.15_Pb(I_0.85_Br_0.15_)_3_ device and PCE of 23.0% was achieved for the champion Cs_0.05_FA_0.90_MA_0.05_Pb(I_0.95_Br_0.05_)_3_ device on 0.159 cm^2^ with negligible hysteresis. Our most stable device retained 96% of its initial efficiency after 2400 h of storage in RH of <30% without any encapsulation. This work provides a promising one‐step antisolvent fabrication route for achieving high‐efficiency and improving stability of perovskite solar cells.

## Experimental Section

##### Precursor Preparation

All materials mentioned were purchased from Greatcell Solar, Alfa Aesar, or Sigma‐Aldrich. More specifically, lead idiode (PbI_2_) from Sigma‐Aldrich, lead bromide (PbBr_2_) from Alfa Aesar, FAI and methylammonium bromide (MABr) from Greatcell Solar, and cesium iodide (CsI) from Alfa Aesar. Solvents used for perovskite precursors include anhydrous DMF and DMSO from Alfa Aesar. The Ac and CB used for antisolvent process were from Sigma‐Aldrich. The acetic acid was dried by the desiccant before use. All other chemicals were used as received without any further purification. The perovskite thin films were made by the composition of mixed halides Cs_0.05_FA_0.80_MA_0.15_Pb(I_0.85_Br_0.15_)_3_. More specifically, the 1 mL precursor (DMF: DMSO = 4:1) contains 1.4 m perovskite.

##### Device Fabrication

The device structure in this research was: FTO/*c*‐TiO_2_/*mp*‐TiO_2_/Cs_0.05_FA_0.80_MA_0.15_Pb(I_0.85_Br_0.15_)_3_ perovskite/Spiro‐OMeTAD/Au. First, the FTO substrate was precleaned by 2% Hellmanex detergent, acetone, isothanol, and ethanol subsequently. The substrates were treated by ultraviolet ozone (UVO) for 15 min. Then the compact TiO_2_ (*c*‐TiO_2_) was deposited by spray pyrolysis (≈50 nm) using 180 × 10^−3^ m titanium diisopropoxide bis(acetylacetonate) solution at 450 °C on the clean FTO glass in ambient atmosphere. After cooling down to room temperature, a 135 mg mL^−1^ of mesoporous TiO_2_ in ethanol solution was spin‐coated at 4000 rpm onto the *c*‐TiO_2_ layer. Then, the substrates were annealed at 105 °C for 10 min followed by sintering at 500 °C for 30 min which results in ≈200 nm in thickness. The substrates were then transferred into nitrogen glove box (O_2_ and H_2_O both bellow 2.0 ppm). For perovskite deposition, a 50 µL 1.48 m Cs_0.05_FA_0.80_MA_0.15_Pb(I_0.85_Br_0.15_)_3_ precursor solution was spin‐coated onto the *m*‐TiO_2_ layer at the speed 2000 rpm for 10 s (acceleration rate at 200 and 4000 rpm for 30 s (acceleration rate at 2000 rpm s^−1^). During the second stage of spin coating (high speed) process, the antisolvent which contains different volumes of Ac was dripped (at 20 s) onto the perovskite surface. The glove box was purged in N_2_ for minimizing solvent residues of Ac and CB. The as‐deposited perovskite films were then left idle for 5 min in a capped petri dish and were subsequently annealed at 100 °C for 10 min, during which the color of the films converted from brown to black. After being cooled down to room temperature, the hole transport solution Spiro‐OMeTAD containing 72.3 mg Spiro‐OMeTAD, 35 µL of a 260 mg mL^−1^ lithium bis(trifluoro‐methylsulphonyl)imide in acetonitrile and 30 µL of 4‐tert‐butylpyridine in 1 mL chlorobenzene was spin‐coated onto the perovskite/*mp*‐TiO_2_/*c*‐TiO_2_/FTO substrate at 3000 rpm for 30 s producing ≈200 nm of Spiro‐OMeTAD layer. Finally, 100 nm thick Au cathode was deposited by thermal vacuum evaporation under 10^−6^ Torr.

##### Characterizations

The current density–voltage (*J–V*) parameters of the devices were characterized by using an NREL calibrated Keithley 2400 digital source meter under simulated AM 1.5G solar irradiation at 100 mW cm^−2^. Note that reverse scan was from *V*_oc_ to *J*_sc_ (forward bias to short circuit, 1.2 to −0.2 V) and forward scan was from *J*_sc_ to *V*_oc_ (short circuit to forward bias, −0.2 to 1.2 V). The EQE measurement was carried out using the PV Measurement QXE7 Spectral Response system with monochromatic light from a xenon arc lamp. The ultraviolet–visible spectroscopy (UV–vis) spectra were measured by a Perkin Elmer model Lambda 1050 instrument. Conventional XRD measurement was conducted using a PANalytical 80 equipment (Empyrean, Cu Ka radiation) at 45 kV and 40 mA. Top‐view and cross‐sectional SEM images were obtained using a field emission SEM (NanoSEM 450). AFM measurements were performed by the Bruker Dimension ICON SPM with a Nanoscope V controller. A platinum–iridium coated AFM tip (SCM‐PIT‐V2, Bruker AFM probes) was used to scan the surface. The c‐AFM was conducted using JEOL JSPM 5400 MKII Environmental Force Microscope using contact mode. During the c‐AFM measurement, a set of *IV* curves were first measured to gauge the conductive behavior of the perovskite. Amplitude modulated KPFM (AM‐KPFM) measurement were performed using the Bruker Dimension ICON SPM with a Nanoscope V controller. A platinum–iridium coated AFM tip (SCM‐PIT‐V2, Bruker AFM probes) was used to scan the surface. Then the perovskite film was scanned with a small applied bias voltage for the actual measurement. Steady‐state PL measurements were conducted using Andor iVac CCD detector (detector temperature was −30 °C). The excitation wavelength of the CW laser was 409 nm and signal was collected under one second exposure time. The TRPL decay for carrier lifetime was measured by a PicoQuant Microtime 200 system. Excitation laser wavelength was 470 nm and a 550 nm long‐path filter was used for measurement. Note that all above measurements were undertaken at room temperature and in ambient conditions. The XPS was carried out with an X‐ray source of monochromated Al Kα (energy 1486.68 eV) using ESCALAB250Xi, Thermo Scientific, UK.

## Conflict of Interest

The authors declare no conflict of interest.

## Author Contributions

Y.L. and J.S. contributed equally to this work. Y.L. fabricated the device and conduct the XRD, SEM, AFM characterization and analysis. J.S. did the 2D GIWAX, PL, and FITR. J.Z. helped with experimental preparation and the use of equipment. M.Z. helped the XRD characterization. J.L. helped set up the whole compact TiO_2_ spray system. Y.C. and J.B. helped with the TRPL characterization. J.Y. helped the GIWAX analysis. Y.Y. did the KPFM and CAFM characterization and analysis. The manuscript was written by Y.L. and revised by A.W.Y.H.‐B. and S.H. through contributions of all authors. The overall project was supervised by S.H., A.W.Y.H.‐B., and M.G.

## Supporting information

Supporting Information

## References

[1] M. A. Green , A. Ho‐Baillie , H. J. Snaith , Nat. Photonics 2014, 8, 506.

[2] J.‐P. Correa‐Baena , M. Saliba , T. Buonassisi , M. Grätzel , A. Abate , W. Tress , A. Hagfeldt , Science 2017, 358, 739. 29123060 10.1126/science.aam6323

[3] G. S. David , P. McMeekin , W. Rehman , G. E. Eperon , M. T. H. Michael Saliba , A. Haghighirad , N. Sakai , L. Korte , B. Rech , M. B. Johnston , L. M. Herz , H. J. Snaith , Science 2017, 351, 151. 10.1126/science.aad5845 26744401

[4] G. E. Eperon , T. Leijtens , K. A. Bush , R. Prasanna , T. Green , J. T.‐W. Wang , D. P. McMeekin , G. Volonakis , R. L. Milot , R. May , A. Palmstrom , D. J. Slotcavage , R. A. Belisle , J. B. Patel , E. S. Parrott , R. J. Sutton , W. Ma , F. Moghadam , B. Conings , A. Babayigit , H.‐G. Boyen , S. Bent , F. Giustino , L. M. Herz , M. B. Johnston , M. D. McGehee , H. J. Snaith , Science 2016, 354, 861. 27856902 10.1126/science.aaf9717

[5] M. M. Lee , J. Teuscher , T. Miyasaka , T. N. Murakami , H. J. Snaith , Science 2012, 338, 643. 23042296 10.1126/science.1228604

[6] S. D. Stranks , G. E. Eperon , G. Grancini , C. Menelaou , M. J. Alcocer , T. Leijtens , L. M. Herz , A. Petrozza , H. J. Snaith , Science 2013, 342, 341. 24136964 10.1126/science.1243982

[7] A. R. M. Abhishek Swarnkar , E. M. Sanehira , B. D. Chernomordik , D. T. Moore , J. A. Christians , J. M. L. Tamoghna Chakrabarti , Science 2016, 354, 92. 27846497 10.1126/science.aag2700

[8] E. M. Sanehira , A. R. Marshall , J. A. Christians , S. P. Harvey , P. N. Ciesielski , L. M. Wheeler , P. Schulz , L. Y. Lin , M. C. Beard , J. M. Luther , Sci. Adv. 2017, 3, eaao4204. 29098184 10.1126/sciadv.aao4204 PMC5659658

[9] M. V. Kovalenko , L. Protesescu , M. I. Bodnarchuk , Science 2017, 358, 745. 29123061 10.1126/science.aam7093

[10] K. Lin , J. Xing , L. N. Quan , F. P. G. de Arquer , X. Gong , J. Lu , L. Xie , W. Zhao , D. Zhang , C. Yan , W. Li , X. Liu , Y. Lu , J. Kirman , E. H. Sargent , Q. Xiong , Z. Wei , Nature 2018, 562, 245. 30305741 10.1038/s41586-018-0575-3

[11] Y. Cao , N. Wang , H. Tian , J. Guo , Y. Wei , H. Chen , Y. Miao , W. Zou , K. Pan , Y. He , H. Cao , Y. Ke , M. Xu , Y. Wang , M. Yang , K. Du , Z. Fu , D. Kong , D. Dai , Y. Jin , G. Li , H. Li , Q. Peng , J. Wang , W. Huang , Nature 2018, 562, 249. 30305742 10.1038/s41586-018-0576-2

[12] J. Luo , X. Wang , S. Li , J. Liu , Y. Guo , G. Niu , L. Yao , Y. Fu , L. Gao , Q. Dong , C. Zhao , M. Leng , F. Ma , W. Liang , L. Wang , S. Jin , J. Han , L. Zhang , J. Etheridge , J. Wang , Y. Yan , E. H. Sargent , J. Tang , Nature 2018, 563, 541. 30405238 10.1038/s41586-018-0691-0

[13] B. Zhao , S. Bai , V. Kim , R. Lamboll , R. Shivanna , F. Auras , J. M. Richter , L. Yang , L. Dai , M. Alsari , X.‐J. She , L. Liang , J. Zhang , S. Lilliu , P. Gao , H. J. Snaith , J. Wang , N. C. Greenham , R. H. Friend , D. Di , Nat. Photonics 2018, 12, 783.

[14] R. H. Friend , D. Di , S. Lilliu , B. Zhao , Sci. Video Protoc. 2019, 1, 1.

[15] W. Yu , F. Li , L. Yu , M. R. Niazi , Y. Zou , D. Corzo , A. Basu , C. Ma , S. Dey , M. L. Tietze , U. Buttner , X. Wang , Z. Wang , M. N. Hedhili , C. Guo , T. Wu , A. Amassian , Nat. Commun. 2018, 9, 5354. 30559392 10.1038/s41467-018-07706-9 PMC6297354

[16] X. Y. Chin , D. Cortecchia , J. Yin , A. Bruno , C. Soci , Nat. Commun. 2015, 6, 7383. 26108967 10.1038/ncomms8383 PMC4491174

[17] S. P. Senanayak , B. Yang , T. H. Thomas , N. Giesbrecht , W. Huang , E. Gann , B. Nair , K. Goedel , S. Guha , X. Moya , C. R. McNeill , P. Docampo , A. Sadhanala , R. H. Friend , H. Sirringhaus , Sci. Adv. 2017, 3, e1601935. 28138550 10.1126/sciadv.1601935 PMC5271592

[18] H. Zhu , Y. Fu , F. Meng , X. Wu , Z. Gong , Q. Ding , M. V. Gustafsson , M. T. Trinh , S. Jin , X. Y. Zhu , Nat. Mater. 2015, 14, 636. 25849532 10.1038/nmat4271

[19] J. H. Park , J. Seo , S. Park , S. S. Shin , Y. C. Kim , N. J. Jeon , H.‐W. Shin , T. K. Ahn , J. H. Noh , S. C. Yoon , C. S. Hwang , S. I. Seok , Adv. Mater. 2015, 27, 4013. 26038099 10.1002/adma.201500523

[20] W. L. Tsai , C. Y. Chen , Y. T. Wen , L. Yang , Y. L. Cheng , H. W. Lin , Adv. Mater. 2019, 31, 1900231. 10.1002/adma.201900231 31020730

[21] J. Choi , J. S. Han , K. Hong , S. Y. Kim , H. W. Jang , Adv. Mater. 2018, 30, 1704002. 10.1002/adma.201704002 29847692

[22] M. A. Green , E. D. Dunlop , D. H. Levi , J. Hohl‐Ebinger , M. Yoshita , A. W. Y. Ho‐Baillie , Prog. Photovoltaics 2019, 27, 565.

[23] P. Gao , M. Grätzel , M. K. Nazeeruddin , Energy Environ. Sci. 2014, 7, 2448.

[24] N. J. Jeon , J. H. Noh , Y. C. Kim , W. S. Yang , S. Ryu , S. I. Seok , Nat. Mater. 2014, 13, 897. 24997740 10.1038/nmat4014

[25] N. K. Noel , A. Abate , S. D. Stranks , E. S. Parrott , V. M. Burlakov , A. Goriely , H. J. Snaith , ACS Nano 2014, 8, 9815. 25171692 10.1021/nn5036476

[26] A. Abate , M. Saliba , D. J. Hollman , S. D. Stranks , K. Wojciechowski , R. Avolio , G. Grancini , A. Petrozza , H. J. Snaith , Nano Lett. 2014, 14, 3247. 24787646 10.1021/nl500627x

[27] Q. Jiang , Y. Zhao , X. Zhang , X. Yang , Y. Chen , Z. Chu , Q. Ye , X. Li , Z. Yin , J. You , Nat. Photonics 2019, 13, 460.

[28] F. Liu , Q. Dong , M. K. Wong , A. B. Djurišić , A. Ng , Z. Ren , Q. Shen , C. Surya , W. K. Chan , J. Wang , A. M. C. Ng , C. Liao , H. Li , K. Shih , C. Wei , H. Su , J. Dai , Adv. Energy Mater. 2016, 6, 1502206.

[29] T. P. Gujar , T. Unger , A. Schonleber , M. Fried , F. Panzer , S. van Smaalen , A. Kohler , M. Thelakkat , Phys. Chem. Chem. Phys. 2017, 20, 605. 29227490 10.1039/c7cp04749e

[30] T. J. Jacobsson , J. P. Correa‐Baena , E. Halvani Anaraki , B. Philippe , S. D. Stranks , M. E. Bouduban , W. Tress , K. Schenk , J. Teuscher , J. E. Moser , H. Rensmo , A. Hagfeldt , J. Am. Chem. Soc. 2016, 138, 10331. 27437906 10.1021/jacs.6b06320

[31] L. Yang , J. Wang , W. W. Leung , ACS Appl. Mater. Interfaces 2015, 7, 14614. 26108296 10.1021/acsami.5b01049

[32] A. Mei , X. Li , L. Liu , Z. Ku , T. Liu , Y. Rong , M. Xu , M. Hu , J. Chen , Y. Yang , M. Gratzel , H. Han , Science 2014, 345, 295. 25035487 10.1126/science.1254763

[33] P. W. Liang , C. Y. Liao , C. C. Chueh , F. Zuo , S. T. Williams , X. K. Xin , J. Lin , A. K. Jen , Adv. Mater. 2014, 26, 3748. 24634141 10.1002/adma.201400231

[34] C.‐H. Chiang , C.‐G. Wu , Nat. Photonics 2016, 10, 196.

[35] J. Huang , S. Tan , P. D. Lund , H. Zhou , Energy Environ. Sci. 2017, 10, 2284.

[36] X. Gong , M. Li , X.‐B. Shi , H. Ma , Z.‐K. Wang , L.‐S. Liao , Adv. Funct. Mater. 2015, 25, 6671.

[37] D. Bi , C. Yi , J. Luo , J.‐D. Décoppet , F. Zhang , S. M. Zakeeruddin , X. Li , A. Hagfeldt , M. Grätzel , Nat. Energy 2016, 1, 16142. 10.1126/science.aaf8060 27284168

[38] F. Zhang , W. Shi , J. Luo , N. Pellet , C. Yi , X. Li , X. Zhao , T. J. S. Dennis , X. Li , S. Wang , Y. Xiao , S. M. Zakeeruddin , D. Bi , M. Gratzel , Adv. Mater. 2017, 29, 1606806. 10.1002/adma.201606806 28240401

[39] F. Wang , W. Geng , Y. Zhou , H.‐H. Fang , C.‐J. Tong , M. A. Loi , L.‐M. Liu , N. Zhao , Adv. Mater. 2016, 28, 9986. 27677653 10.1002/adma.201603062

[40] Y. Yang , S. Feng , M. Li , F. Li , C. Zhang , Y. Han , L. Li , J. Yuan , L. Cao , Z. Wang , B. Sun , X. Gao , Nano Energy 2018, 48, 10.

[41] F. Li , J. Yuan , X. Ling , Y. Zhang , Y. Yang , S. H. Cheung , C. H. Y. Ho , X. Gao , W. Ma , Adv. Funct. Mater. 28, 2018, 1706377.

[42] E. H. Jung , N. J. Jeon , E. Y. Park , C. S. Moon , T. J. Shin , T. Y. Yang , J. H. Noh , J. Seo , Nature 2019, 567, 511. 30918371 10.1038/s41586-019-1036-3

[43] J. J. Yoo , S. Wieghold , M. Sponseller , M. Chua , S. N. Bertram , N. T. P. Hartono , J. Tresback , E. Hansen , J.‐P. Correa‐Baena , V. Bulovic , T. Buonassisi , S. S. Shin , M. G. Bawendi , Energy Environ. Sci. 2019, 12, 2192.

[44] W. Zhang , M. Saliba , D. T. Moore , S. K. Pathak , M. T. Horantner , T. Stergiopoulos , S. D. Stranks , G. E. Eperon , J. A. Alexander‐Webber , A. Abate , A. Sadhanala , S. Yao , Y. Chen , R. H. Friend , L. A. Estroff , U. Wiesner , H. J. Snaith , Nat. Commun. 2015, 6, 6142. 25635571 10.1038/ncomms7142

[45] Q. Zhao , G. R. Li , J. Song , Y. Zhao , Y. Qiang , X. P. Gao , Sci. Rep. 2016, 6, 38670. 27934924 10.1038/srep38670 PMC5146662

[46] M. E. Pampulha , M. C. Loureiro‐Dias , Appl. Microbiol. Biotechnol. 1989, 31, 547.

[47] K. A. Bush , N. Rolston , A. Gold‐Parker , S. Manzoor , J. Hausele , Z. J. Yu , J. A. Raiford , R. Cheacharoen , Z. C. Holman , M. F. Toney , R. H. Dauskardt , M. D. McGehee , ACS Energy Lett. 3, 2018, 1225.

[48] S. Braunger , L. E. Mundt , C. M. Wolff , M. Mews , C. Rehermann , M. Jošt , A. Tejada , D. Eisenhauer , C. Becker , J. A. Guerra , E. Unger , L. Korte , D. Neher , M. C. Schubert , B. Rech , S. Albrecht , J. Phys. Chem. C 2018, 122, 17123.

[49] A. Bercegol , F. J. Ramos , A. Rebai , T. Guillemot , J.‐B. Puel , J.‐F. Guillemoles , D. Ory , J. Rousset , L. Lombez , J. Phys. Chem. C 2018, 122, 23345.

[50] B.‐E. Cohen , S. Aharon , A. Dymshits , L. Etgar , J. Phys. Chem. C 2016, 120, 142.

[51] Y. Cho , A. M. Soufiani , J. S. Yun , J. Kim , D. S. Lee , J. Seidel , X. Deng , M. A. Green , S. Huang , A. W. Y. Ho‐Baillie , Adv. Energy Mater. 2018, 8, 1703392.

[52] M. Saliba , T. Matsui , J. Y. Seo , K. Domanski , J. P. Correa‐Baena , M. K. Nazeeruddin , S. M. Zakeeruddin , W. Tress , A. Abate , A. Hagfeldt , M. Gratzel , Energy Environ. Sci. 2016, 9, 1989. 27478500 10.1039/c5ee03874j PMC4936376

[53] M. Abdi‐Jalebi , Z. Andaji‐Garmaroudi , S. Cacovich , C. Stavrakas , B. Philippe , J. M. Richter , M. Alsari , E. P. Booker , E. M. Hutter , A. J. Pearson , S. Lilliu , T. J. Savenije , H. Rensmo , G. Divitini , C. Ducati , R. H. Friend , S. D. Stranks , Nature 2018, 555, 497. 29565365 10.1038/nature25989

[54] D. T. Moore , H. Sai , K. W. Tan , D. M. Smilgies , W. Zhang , H. J. Snaith , U. Wiesner , L. A. Estroff , J. Am. Chem. Soc. 2015, 137, 2350. 25625616 10.1021/ja512117e

[55] J. Peng , J. I. Khan , W. Liu , E. Ugur , T. Duong , Y. Wu , H. Shen , K. Wang , H. Dang , E. Aydin , X. Yang , Y. Wan , K. J. Weber , K. R. Catchpole , F. Laquai , S. De Wolf , T. P. White , Adv. Energy Mater. 2018, 8, 1801208.

