# Supplementary material for: Acetic Acid Assisted Crystallization Strategy for High Efficiency and Long‐Term Stable Perovskite Solar Cell
Source: Adv Sci (Weinh). 2020 Jan 23;7(5):1903368. doi: 10.1002/advs.201903368 (PMC7055551; doi:10.1002/advs.201903368)
Supplement: Supplementary file 1 — Supporting Information [file ADVS-7-1903368-s001.pdf]

Copyright WILEY-VCH Verlag GmbH & Co. KGaA, 69469 Weinheim, Germany, 2020.

ADVANCED SCIENCE
Open Access

Supporting Information

for *Adv. Sci.,* DOI: 10.1002/advs.201903368

Acetic Acid Assisted Crystallization Strategy for High Efficiency and Long-Term Stable Perovskite Solar Cell

Yong Li, Junwei Shi, Jianghui Zheng, Jueming Bing, Jianyu Yuan, Yongyoon Cho, Shi Tang, Meng Zhang, Yin Yao, Cho Fai Jonathan Lau, Da Seul Lee, Chwenhaw Liao, Martin A. Green, Shujuan Huang, Wanli Ma, and Anita W. Y. Ho-Baillie**

Copyright WILEY-VCH Verlag GmbH & Co. KGaA, 69469 Weinheim, Germany, 2019.

Supplementary Information

Acetic acid assisted crystallization strategy for high efficiency and long-term stable perovskite solar cell

Yong Li, Junwei Shi, Jianghui Zheng, Jueming Bing, Jianyu Yuan, Yongyoon Cho, Shi Tang, Meng Zhang, Yin Yao, Cho Fai Jonathan Lau, Da Seul Lee, Chwenhaw Liao, Martin A. Green, Shujuan Huang, Wanli Ma, Anita W. Y. Ho-Baillie**

Y. Li, Dr. J. Zheng, J. Bing, Y. Cho, S. Tang, Dr. M. Zhang, Dr. C. F. J. Lau, D. S. Lee, C. Liao, Prof. M. A. Green, Assoc. Prof. A. W. Y. Ho-Baillie
Australian Centre for Advanced Photovoltaics, School of Photovoltaic and Renewable Energy Engineering, University of New South Wales, Sydney 2052, Australia
E-mail: a.ho-baillie@unsw.edu.au

Assoc. Prof. S. Huang, Assoc. Prof. A. W. Y. Ho-Baillie
School of Engineering, Macquarie University, Sydney 2109, Australia

J. Shi, J. Yuan, Prof. W. Ma
Jiangsu Key Laboratory for Carbon-Based Functional Materials and Devices, Institute of Functional Nano & Soft Materials (FUNSOM), Soochow University, Suzhou 215123, China
E-mail: wlma@suda.edu.cn

Dr. Y. Yao
Electron Microscope Unit, Mark Wainwright Analytical Centre, The University of New South Wales, Sydney, New South Wales, 2052, Australia

Keywords: photovoltaic, perovskites, anti-solvent engineering, high efficiency, stability

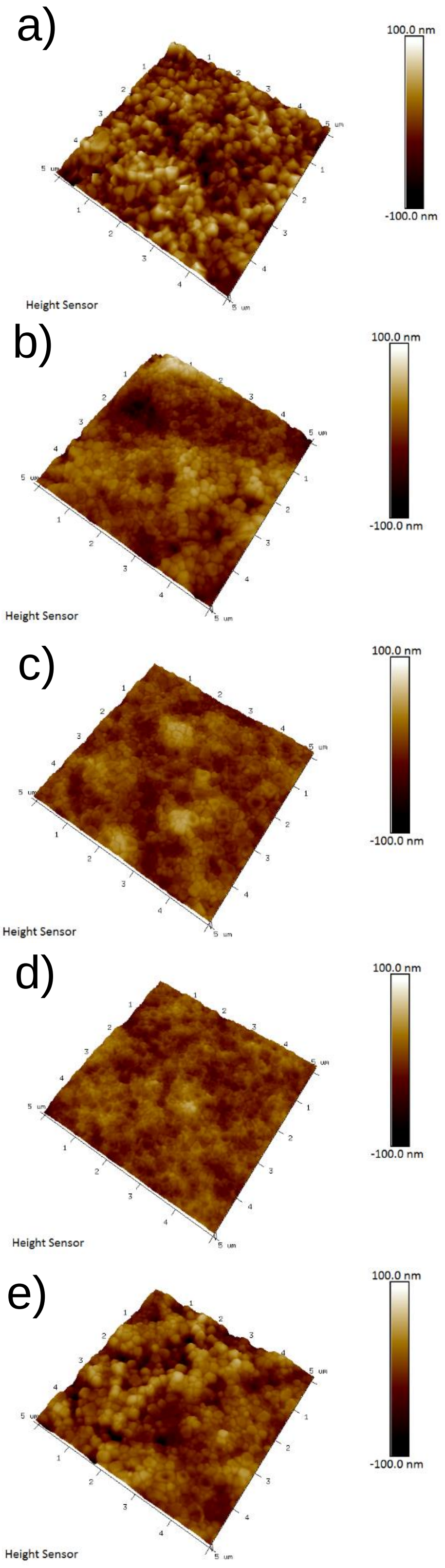

Figure S1. The atomic force microscopy (AFM, 3D) of the perovskite films fabricated by Ac antisolvent treatment. a) without Ac, b) with Ac 2, c) with Ac 5, d) with Ac 8, and e) with Ac 10.

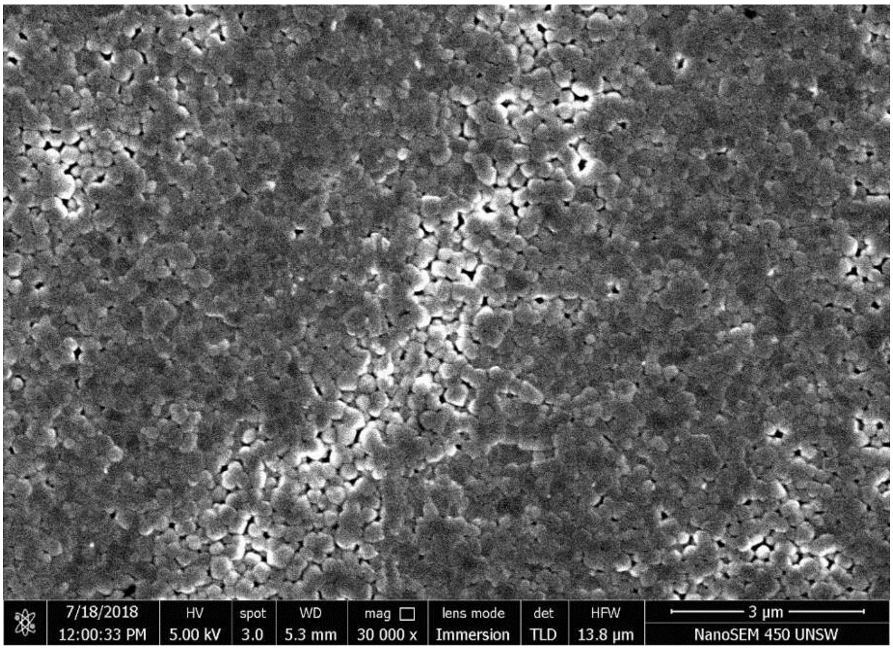

Figure S2. SEM of the perovskite film treated with Ac 10. The bright area was possibly corroded by Ac.

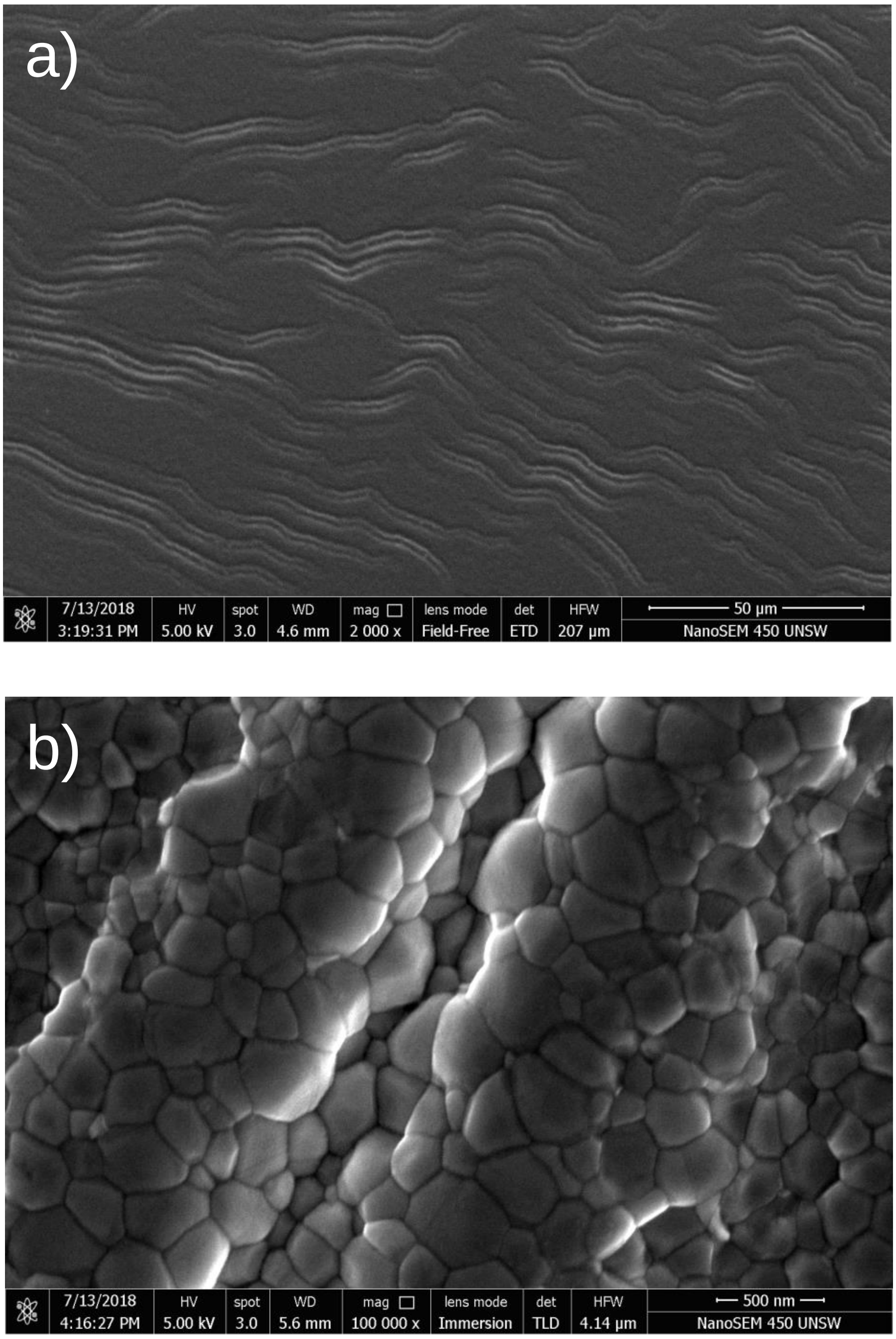

Figure S3. Typical wrinkle morphology. SEM of the perovskite film with Ac 8 showing "wrinkle" morphology at (a) low and (b) high magnifications. Scale bars are 50 µm in (a) and 500 nm in (b).

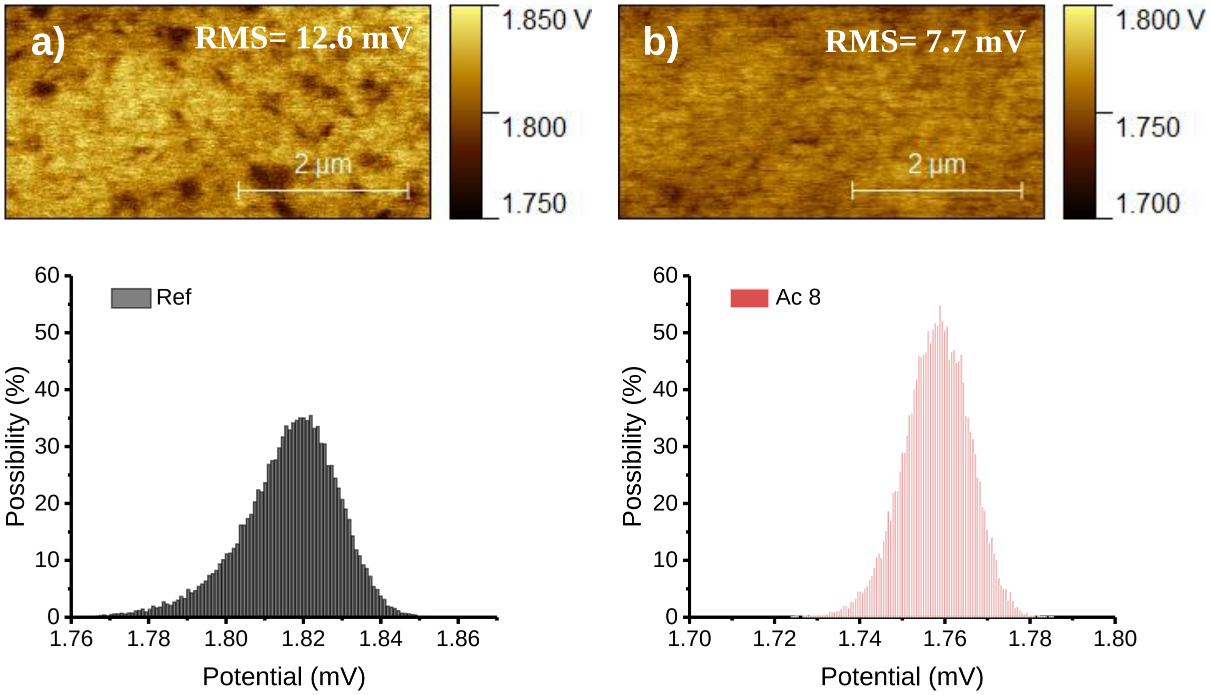

Figure S4. Kelvin probe force microscopy (KPFM) showing surface potentials and their distribution of the reference (a) and Ac 8 treated (b) perovskite films measured in the dark.

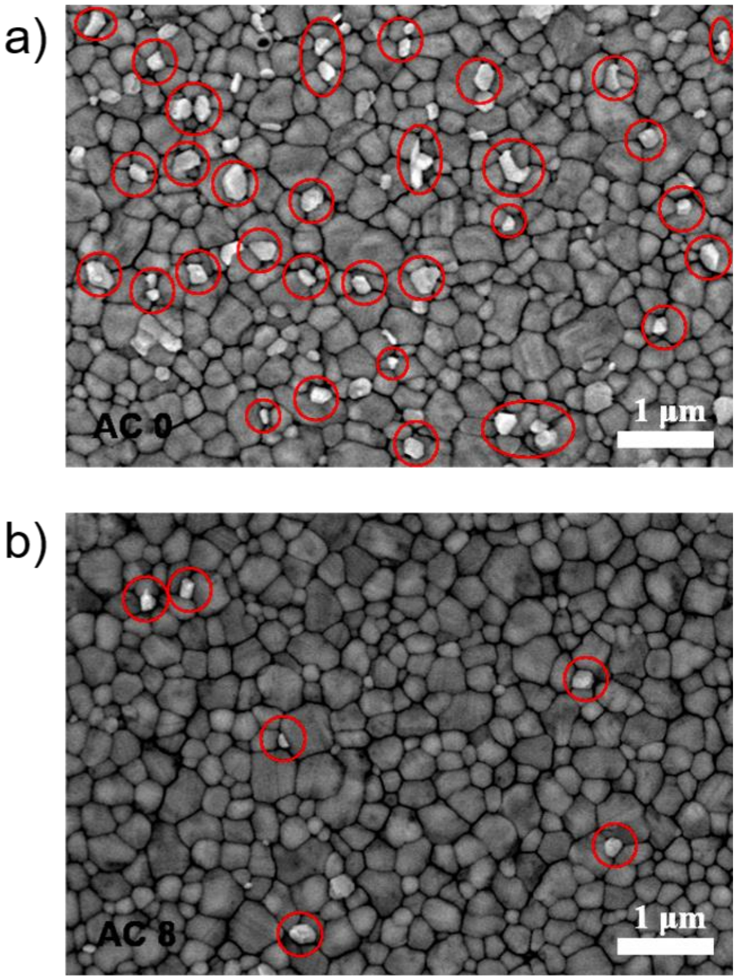

Figure S5. Backscattered SEM (BS-SEM) images. a) Ac 0 and (b) Ac 8 perovskite films. PbI_2 residuals are marked by red circles.

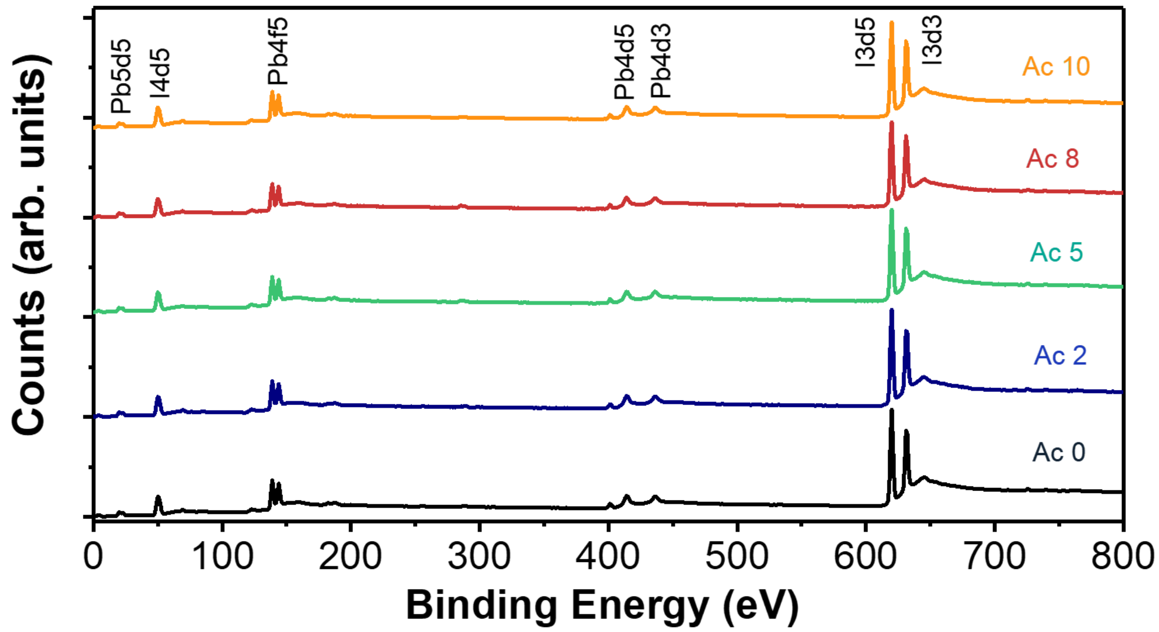

Figure S6. XPS spectra of the perovskite films with different levels of Ac treatment. XPS Showing negligible change indicating no change in perovskite composition.

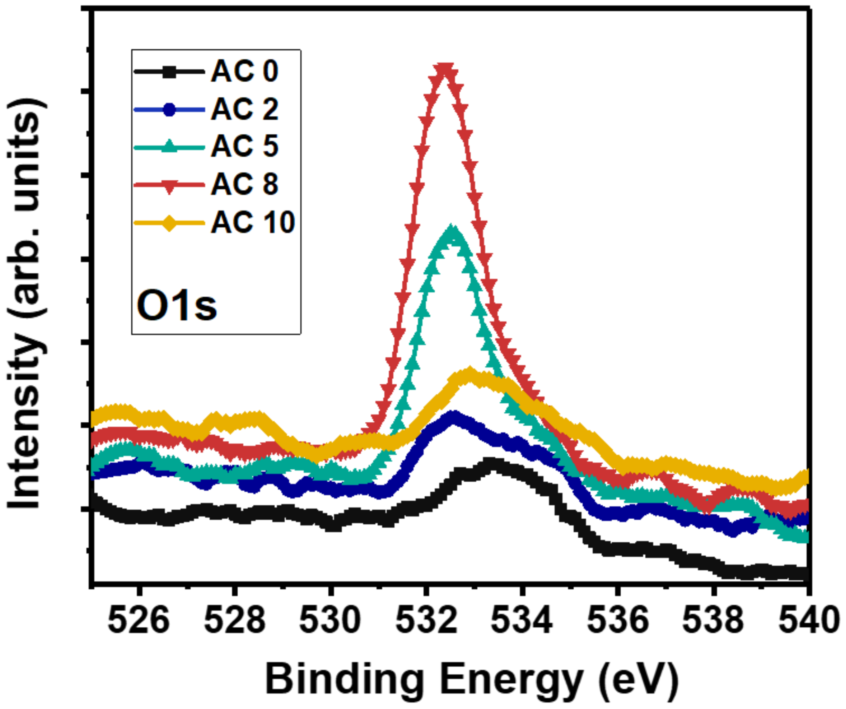

Figure S7. XPS spectra of O 1s for the perovskite films with different levels of Ac treatment.

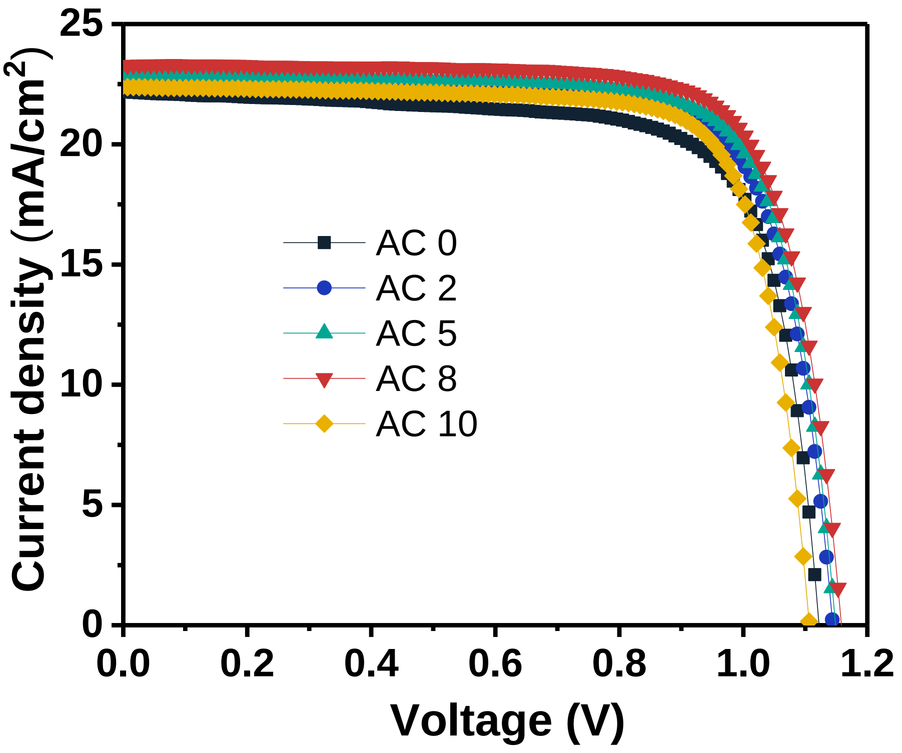

Figure S8. Typical *J-V* curves of devices with different levels of Ac treatment.

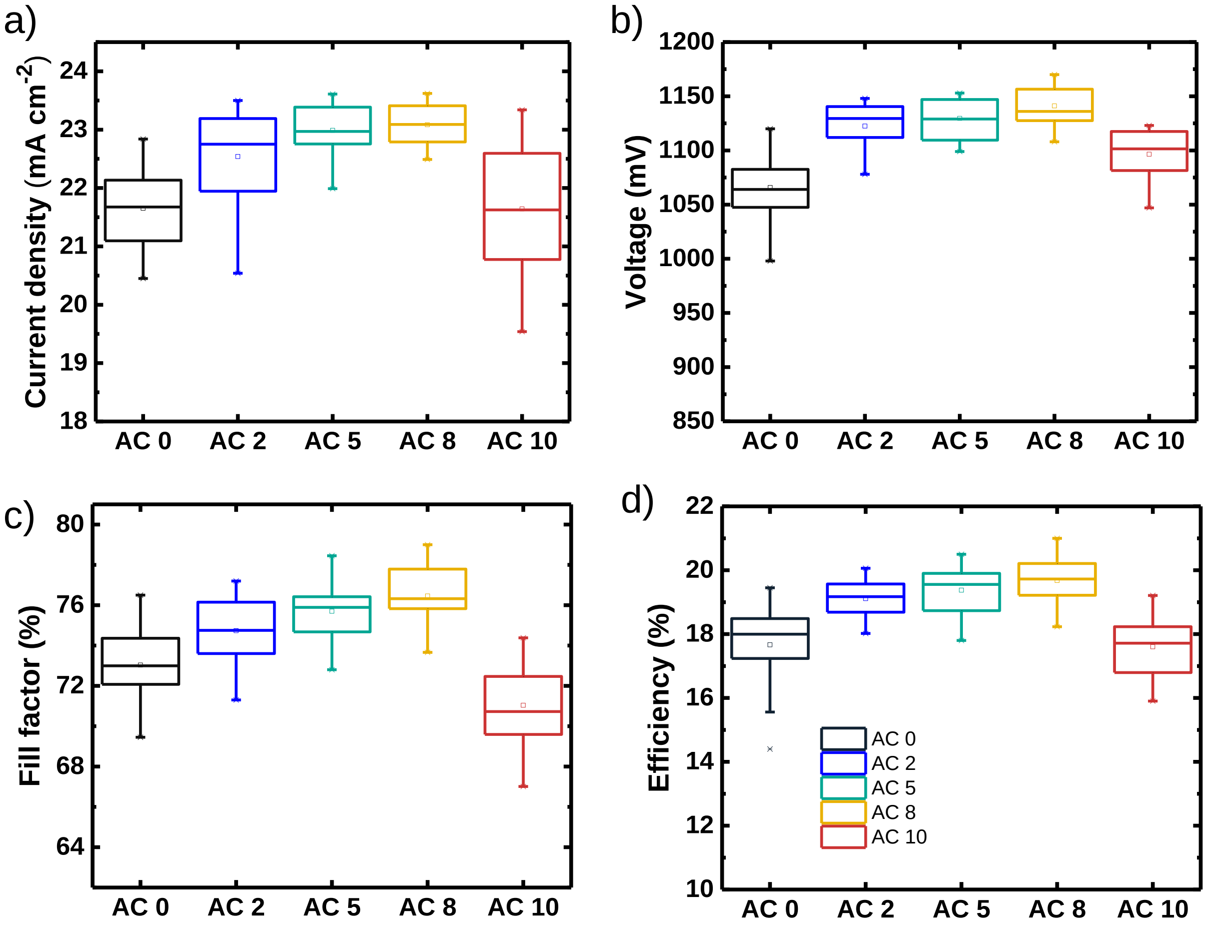

Figure S9. Distribution of perovskite photovoltaic parameters. a) power conversion efficiency (PCE), b) open circuit voltage (V_{oc}), c) short circuit current density (J_{sc}) and d) Fill factor (FF) of reference and Ac treated cells.

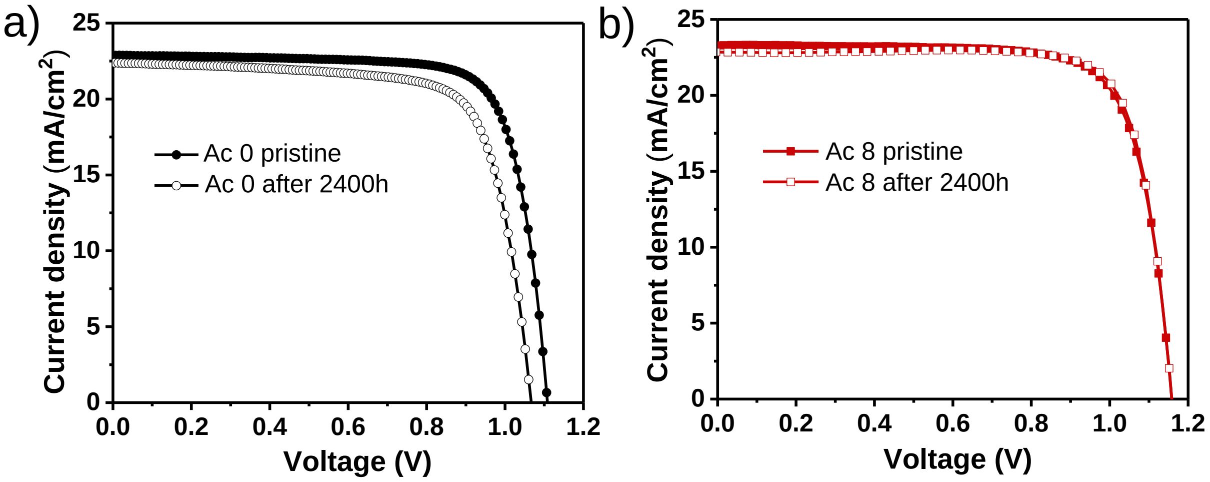

Figure S10. Reference sample Ac 0 and best performance sample Ac 8 device after storage for 100 days for stability test in ambient environment with controlled RH<30%.

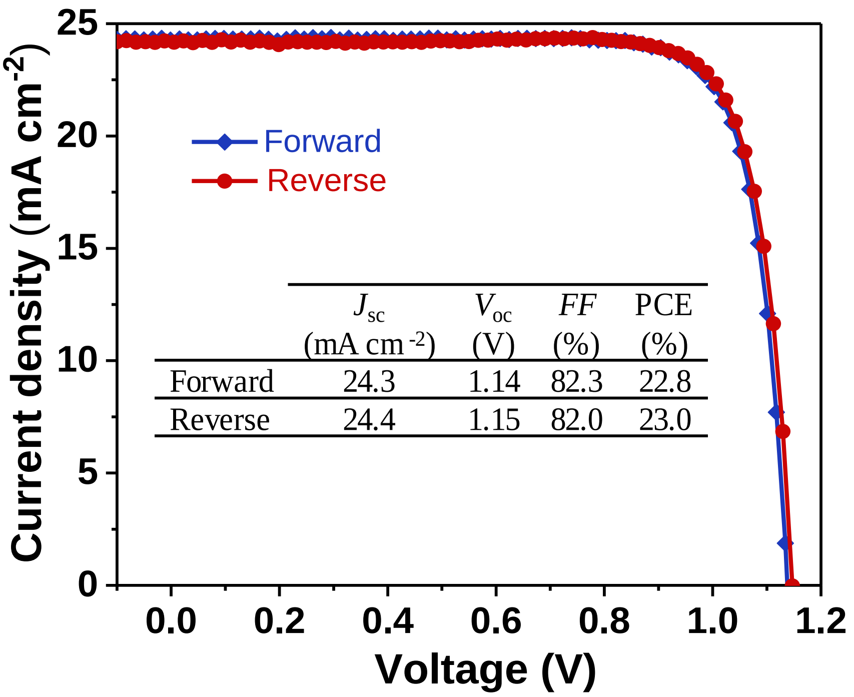

Figure S11. Champaign device efficiency for $Cs_{0.05}FA_{0.90}MA_{0.05}Pb(I_{0.95}Br_{0.05})_3$ perovskite solar cell.

Table S1. Summarize of the TRPL lifetime. For the perovskite films with different volume of Ac treatment. Notice the perovskite was spin-coated on the glass substrate.

	**AC 0**	**AC 2**	**AC 5**	**AC 8**	**AC 10**
τ_{eff} (ns)	498	756	782	853	350

Table S2. The ratios of I: Pb in $Cs_{0.05}FA_{0.80}MA_{0.15}Pb(I_{0.85}Br_{0.15})_3$ perovskite film with different Ac treatment.

**AC 0**	**AC 2**	**AC 5**	**AC 8**	**AC 10**
3.25	3.27	3.18	3.20	3.26

Table S3. Parameters of the $Cs_{0.05}FA_{0.80}MA_{0.15}Pb\ (I_{0.85}Br_{0.15})_3$ perovskite device using different levels of Ac treatments.

	J_{sc} (mA cm^{-2})	V_{oc} (mV)	FF (%)	PCE (%)
AC 0	22.9	1108	75.3	19.1
AC 2	22.9	1146	75.1	19.7
AC 5	23.2	1150	75.6	20.2
AC 8	23.4	1162	78.4	21.3
AC 10	22.7	1123	73.1	18.6
